# The puzzling regulation of the interferon signaling system by the p53 tumor suppressor protein

**DOI:** 10.1007/s00018-025-05763-0

**Published:** 2025-06-13

**Authors:** Agnieszka Będzińska, Barbara Łasut-Szyszka, Małgorzata Krześniak, Agnieszka Gdowicz-Kłosok, Marek Rusin

**Affiliations:** Center for Translational Research and Molecular Biology of Cancer, Maria Skłodowska-Curie National Research Institute of Oncology, Gliwice Branch, ul. Wybrzeże Armii Krajowej 15, 44-101 Gliwice, Poland

**Keywords:** NK-92, FAS ligand, Innate immunity, Pyroptosis, IFITM3, OAS1

## Abstract

The p53 tumor suppressor exhibits antiviral activity. The viral replication is also inhibited by interferons (IFNs), cytokines that regulate immune genes via STAT transcription factors. The best studied interferons belong to the type I (e.g., IFNα1) and type II (IFNγ) groups. IFNα1 and IFNγ induce the phosphorylation of STAT1 at Tyr701. Previously, we reported that p53 activates SOCS1, a negative regulator of STAT1 phosphorylation. Based on this, we hypothesized that p53, by activating SOCS1, reduces the phosphorylation of STAT1 and attenuates the activation of genes stimulated either by IFNα1 or IFNγ. To test this hypothesis, we exposed p53-proficient and p53-deficient cells to p53 activators along with either IFNα1 or IFNγ. We then assessed STAT1 phosphorylation and the expression of interferon-regulated genes. Strong p53 activation reduced the STAT1 phosphorylation at Tyr701; however, it did not decrease the expression of most of the tested interferon-stimulated genes. On the contrary, IFNγ synergized with p53 to enhance *CASP1*, *IFIT1* and *IFIT3* expression. We conclude that the interactions between p53 and interferon-activated pathways are more complicated than initially expected, and their cooperation deserves further investigation. Moreover, we found that *SOCS1* can be either up- or down-regulated by p53 depending on cell type and stress conditions.

**Supplementary Information:**

The online version contains supplementary material available at 10.1007/s00018-025-05763-0.

## Introduction

The p53 protein encoded by *TP53* is best known for its ability to inhibit the cell cycle and activate apoptosis [1]. However, the p53 protein not only functions as a tumor suppressor but also as an antiviral factor, for example by stimulating the expression of innate and adaptive immunity genes. For this reason, p53 is a frequent target of inactivation by proteins encoded by viruses [2]. Notably, p53 was discovered as a protein bound and inactivated by the large T antigen of the SV40 virus [3]. Furthermore, p53 is a transcription regulator that directly activates at least several hundred genes [4]. There is also a large group of genes repressed by p53; however, this repression occurs indirectly through the activation of the gene encoding the p21 protein, which inhibits cyclin-dependent kinases, promoting the formation of repressive complexes on cell cycle genes [5]. Interestingly, p53 activates the expression of many genes, which are also stimulated by interferons [6].

Interferons are cytokines involved in both innate and adaptive immunity and playing a major role in the defense against viruses and bacteria. Interferons are strong activators of gene expression. Their activity is mediated by transcription factors from the STAT family. There are three types of interferons, each of which signals through different receptors. The best-studied interferons are interferons-α (e.g., IFNα1), interferon-β (IFNβ) (type I) and interferon-γ (IFNγ, type II). IFNα1 can be secreted by wide-range of cells, whereas IFNγ is secreted only by T cells and natural killer cells. The activity of type-I interferons and interferon-γ critically depends on the STAT1 transcription factor. The binding of type-I interferons to their cognate receptor induces their dimerization, which in turn activates two kinases, JAK1 and TYK2. Activated kinases phosphorylate interferon receptors on target tyrosine residues, which creates a docking site for STAT1 and STAT2 transcription regulators, which become phosphorylated on critical tyrosine residues, such as Tyr701 on STAT1 and Tyr690 on STAT2. Phosphorylated STAT1 and STAT2 bind to the IRF9 protein and, as a trimeric complex known as interferon-stimulated gene factor 3 (ISGF3), bind to the DNA sequence known as the interferon-stimulated response element (ISRE) and activate a set of interferon-stimulated genes (ISGs). IFNγ binds to its receptor and induces the phosphorylation of STAT1 at Tyr701. Phosphorylated STAT1 forms a homodimer known as gamma-activated factor (GAF) and activates its set of genes. The general term interferon-stimulated genes is slightly misleading because type I- and type II-interferons activate different but overlapping sets of genes [7].

The signaling pathways activated by type-I interferons or by interferon-γ are negatively regulated by various proteins. One of them is the SOCS1, which prevents STAT1 phosphorylation. The *SOCS1* gene is activated by interferon-γ and is an important element of the negative feedback loop in this signaling pathway [8, 9]. Previously, we reported that the *SOCS1* gene is positively regulated by p53. We tested the regulation of SOCS1 by p53 because the treatment of cells with a combination of p53 activators actinomycin D and nutlin-3a (A + N) was associated with the reduced phosphorylation of STAT1 at Tyr701 initiated by IFNα1. The logical explanation for this observation is that p53 negatively regulates the phosphorylation of STAT1 at Tyr701. We found that in p53-deficient cells, the activation of *SOCS1* is reduced, suggesting that p53 promotes its expression [10]. Thus, on the basis of this observation, we hypothesized that strong activation of p53 can attenuate the expression of genes regulated by phosphorylated STAT1, e.g., genes activated by either interferon-α1 or interferon-γ. We initiated this study to test this hypothesis.

## Results

### p53 can either activate or repress *SOCS1* expression depending on the cell line

**Fig. 1:**
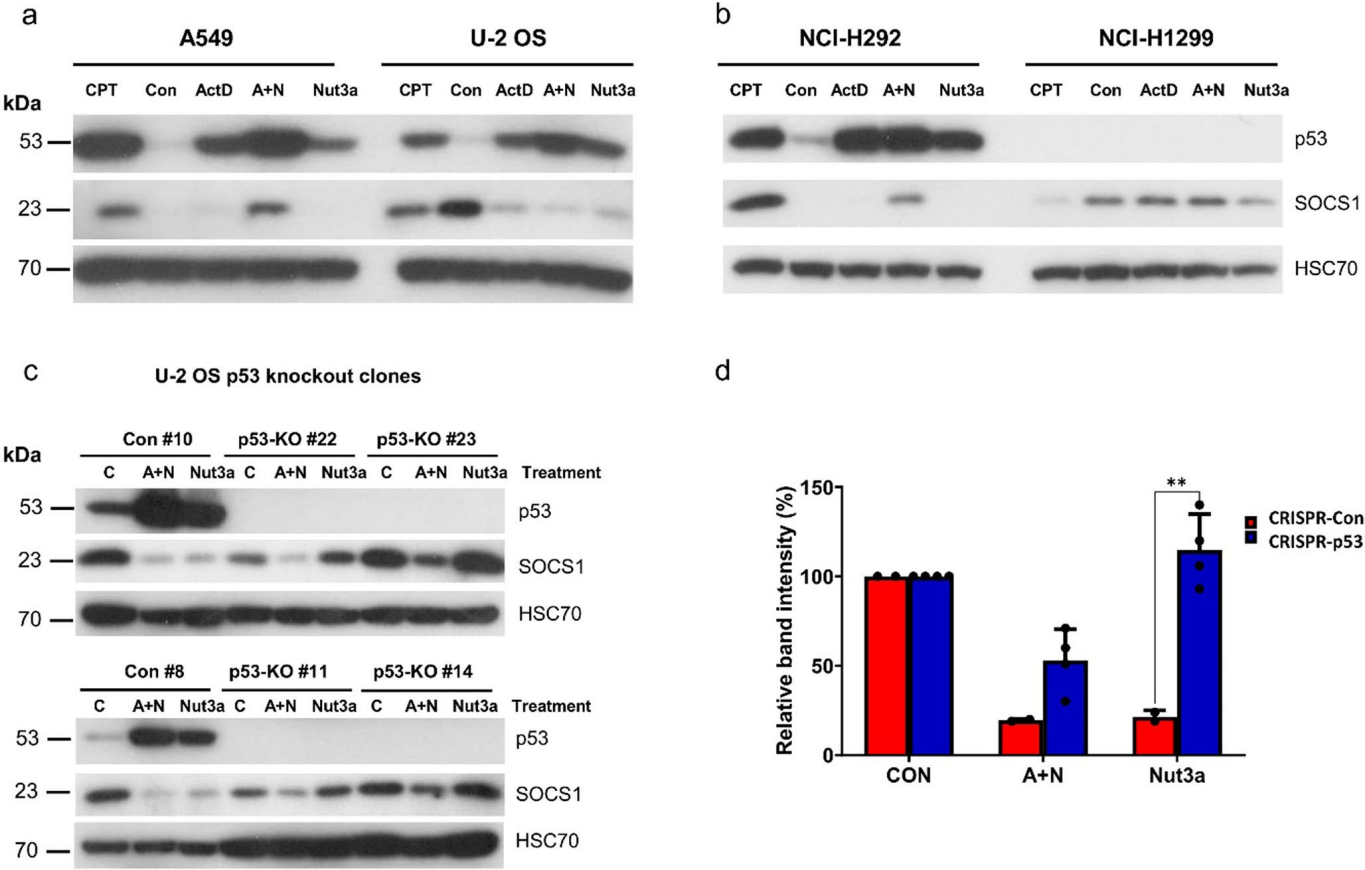
The expression of SOCS1 in response to treatment with actinomycin D and nutlin-3a is regulated in a cell-specific manner. (**a**, **b**) Levels of p53, SOCS1 and the loading control (HSC70) in the indicated cell lines exposed to actinomycin D (ActD), nutlin-3a (Nut3a), or both compounds that act together (A + N) or camptothecin (CPT) for 48 h. Control cells (Con) were mock-treated. (**c**) Levels of p53, SOCS1 and the loading control in p53-proficient (Con) and p53-knockout clones (p53-KO) of the U-2 OS cell line exposed to A + N, nutlin-3a (Nut3a) or mock-treated (C) for 48 h. # indicates the clone number. (**d**) Densitometric analysis of SOCS1 bands presented on panel c, normalized to intensity of HSC70 bands. The SOCS1 band intensity data from individual clones presented on panel c were considered as a separate experiments and used to prepare this graph. The intensity of band in cells growing in control conditions (either p53-proficient or p53-knockout cells – CRISPR-Con, CRISPR-p53, respectively) was set to 100%. The statistical significance of the indicated difference in expression was determined using unpaired t test (Holm-Šídák method, ** *p* < 0.01)

In earlier work, we demonstrated that *SOCS1* gene is induced by p53 in A549 cells – the knockdown of p53 significantly weakened *SOCS1* expression [10]. Activation of *SOCS1* was associated with diminished phosphorylation of STAT1 induced by IFNα1 [10]. In the current project, we tested whether *SOCS1* can be induced by p53 activators in other cell lines. For the first experiment we selected U-2 OS cells because they are frequently employed in transcriptomic studies to search for p53-regulated genes [4]. We exposed these cells in parallel with A549 cells to actinomycin D, nutlin-3a, their combination (A + N) or to camptothecin (Fig. 1a). As expected, in A549 cells, SOCS1 expression was upregulated by camptothecin (CPT) and by A + N. However, in U-2 OS cells, the results were surprisingly different. First, we detected strong constitutive expression of SOCS1. Second, following exposure to actinomycin D, nutlin-3a or A + N, SOCS1 protein was significantly downregulated. These findings suggest that the activation of p53 leads to the repression of constitutively expressed *SOCS1*. In another cell line with wild-type p53 - NCI-H292, the expression pattern of SOCS1 resembled A549 cells (Fig. 1b). Our transcriptomic analysis [6] also found strong upregulation of *SOCS1* mRNA in other cell lines with wild-type p53 exposed to A + N (A375 – melanoma and NCI-H460 – lung cancer). In p53-null cells, i.e., NCI-H1299, the expression of SOCS1 was easily detectable in control condition but was not upregulated following any treatment (Fig. 1b). High-throughput data analyzed by Fischer et al. [4] demonstrated that *SOCS1* can be activated in these cells by ectopically expressed p53.

To test the hypothesis that p53 is responsible for *SOCS1* repression in U-2 OS cells, we prepared via CRISPR/Cas9 technology the p53 knockout clones. Two control clones and four knockout clones were exposed to A + N or nutlin-3a (Fig. 1c). In the control clones, both treatment modalities led to strong repression of SOCS1, as in the parental cell line. However, in all p53 knockout clones, nutlin-3a lost the ability to repress SOCS1. To show it quantitatively, we performed densitometric analysis of SOCS1 bands from blots presented on panel c. The graph (Fig. 1d) confirms the conclusions of the visual inspection of Fig. 1c.

**Fig. 2:**
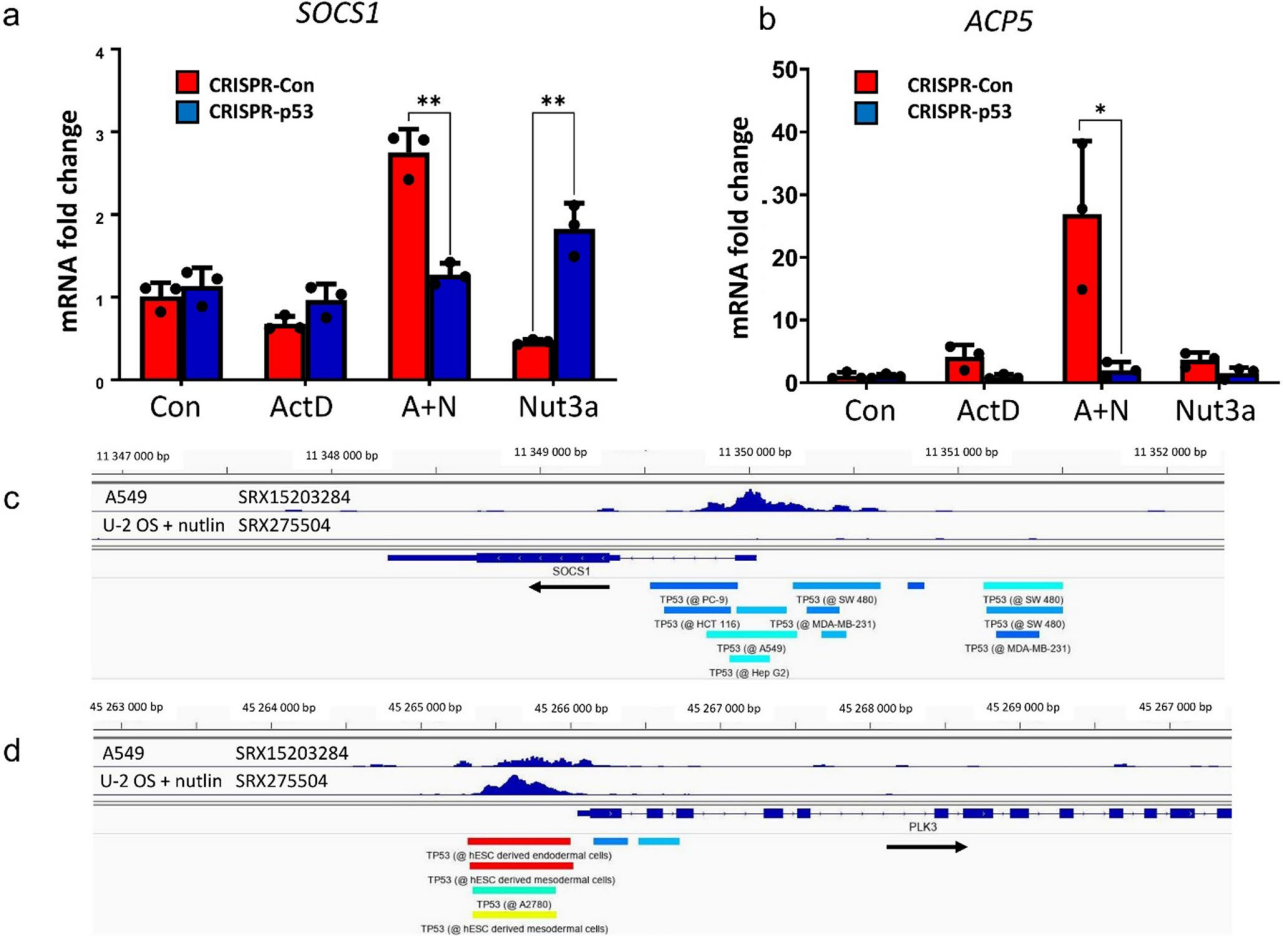
Expression of *SOCS1* mRNA in U-2 OS cells is downregulated in p53-dependent manner. (**a**) The fold-change of *SOCS1* mRNA measured by semi-quantitative RT-PCR in three biological repeats in p53-proficient clone (#8) and p53-deficient clone (#14) treated as indicated with actinomycin D (ActD), A + N or nutlin-3a (Nut3a). The statistical significance of the indicated differences in expression was determined using unpaired t test (Holm-Šídák method, ** *p* < 0.01). (**b**) The fold-change of *ACP5* mRNA measured by semi-quantitative RT-PCR in three biological repeats in p53-proficient clone (#8) and p53-deficient clone (#14). To test statistical significance we employed unpaired t test with Welch correction (* *p* < 0.05). (**c**) Genome Browser (IGV) views of p53 ChIP-Seq peak near the transcription start site of the *SOCS1* gene. Using the ChIP-Atlas tool [11], we imported publicly available coverage tracks from two ChIP-Seq experiments aimed at finding p53 binding sites in the A549 cells growing in control conditions (sample ID SRX15203284) and U-2 OS cells exposed to nutlin (sample ID SRX275504). The location of ChIP-Seq peaks identified by ChIP-Atlas in other samples (not displayed as graph) are shown by horizontal bars at the bottom of the panel. The black arrow shows the direction of gene transcription. (**d**) The p53 ChIP-Seq peaks identified in samples as above upstream transcription start site of *PLK3* gene, which is commonly activated by p53. The horizontal bars show the location of p53 ChIP-Seq peaks in 4 out of more than 100 samples displaying p53 binding in this region. The rest was removed for the sake of picture clarity

To find out whether the downregulation of *SOCS1* in U-2 OS cells occurs via transcriptional or post-transcriptional level, we performed semi-quantitative RT-PCR analysis of *SOCS1* in one p53 proficient clone (#8) and in one p53 knockout clone (#14). The result is presented on Fig. 2a. The mRNA level of *SOCS1* is downregulated in p53-proficient control clone exposed to nutlin-3a. In p53-knockout clone nutlin-3a does not repress *SOCS1* mRNA. Thus, nutlin-3a conspicuously repressed SOCS1 at both protein and mRNA level and this downregulation is governed by p53. Other, newly discovered p53-regulated gene – *ACP5* [6] exhibits a typical pattern of p53-activated genes—upregulation by actinomycin D or nutlin-3a, a synergistic effect of these two substances, and reduced expression in p53-knockout cells (Fig. 2b).

Remarkably, the observed *SOCS1* expression pattern in U-2 OS cells is consistent with high-throughput data analyzed by Fischer et al. [4], who found that *SOCS1* was repressed in all four studies analyzing gene expression in U-2 OS cells exposed to nutlin-3a. The possibility that a gene can be both activated and repressed by p53 may partially explain inconsistencies among different high-throughput studies on p53’s role in gene regulation. The examples are *SOCS1* and *IFI16* genes [4].

The downregulation of SOCS1 protein by A + N (Fig. 1a, c and d) sharply contrasts with the lack of repression of its mRNA (in p53-proficient clone it is even slightly upregulated). Thus, the suppression of SOCS1 protein by A + N in U-2 OS cells occurs at posttranscriptional level and to a large part is p53-independent because it takes place in both p53-proficient and p53-deficient cells (Fig. 1c and d).

To start to uncover the mechanism of *SOCS1* regulation by p53 in A549 and in U-2 OS cell lines we browsed, using ChIP-Atlas tool [11], the publically available p53 ChIP-Seq data bases. We found a data set with the p53 ChIP-Seq peak near the *SOCS1* transcription start site in the A549 cell line (Fig. 2c). In U-2 OS cells exposed to nutlin there is no p53 ChIP-Seq peak within or near *SOCS1* in any of the data sets (27 February 2025). As an example, we presented on Fig. 2c the data from sample SRX275504. In contrast to *SOCS1*, the same samples of A549 and U-2 OS cells show the p53 ChIP-Seq peak near the transcription start site of *PLK3* (Fig. 2d), which is well-known p53-activated gene [4]. Thus, p53 can bind within promoter sequence of *SOCS1* in A549 cells, however there is no evidence of p53 binding near *SOCS1* in U-2 OS cells in any of the accessible data bases. Therefore, the available evidence presented in our earlier experiments [10] and in this paper suggests that *SOCS1* is directly activated by p53 in A549 cells (and also in other cell lines) exposed to A + N, whereas in U-2 OS cells, p53 activated by nutlin-3a suppresses this gene, but it probably happens indirectly because p53 does not bind in vicinity of *SOCS1* in this cell line. This is consistent with the model according to which p53 is only the activator of gene transcription and when it suppresses genes it does it indirectly by activating various mechanisms of gene silencing [12]. What is new and very important in our experiment is the observation that p53 can play double role (activation or suppression) in regulation of the same gene depending on cell type and stress conditions.

### Treatment with A + N reduces the phosphorylation of STAT1 induced by IFNα1

**Fig. 3:**
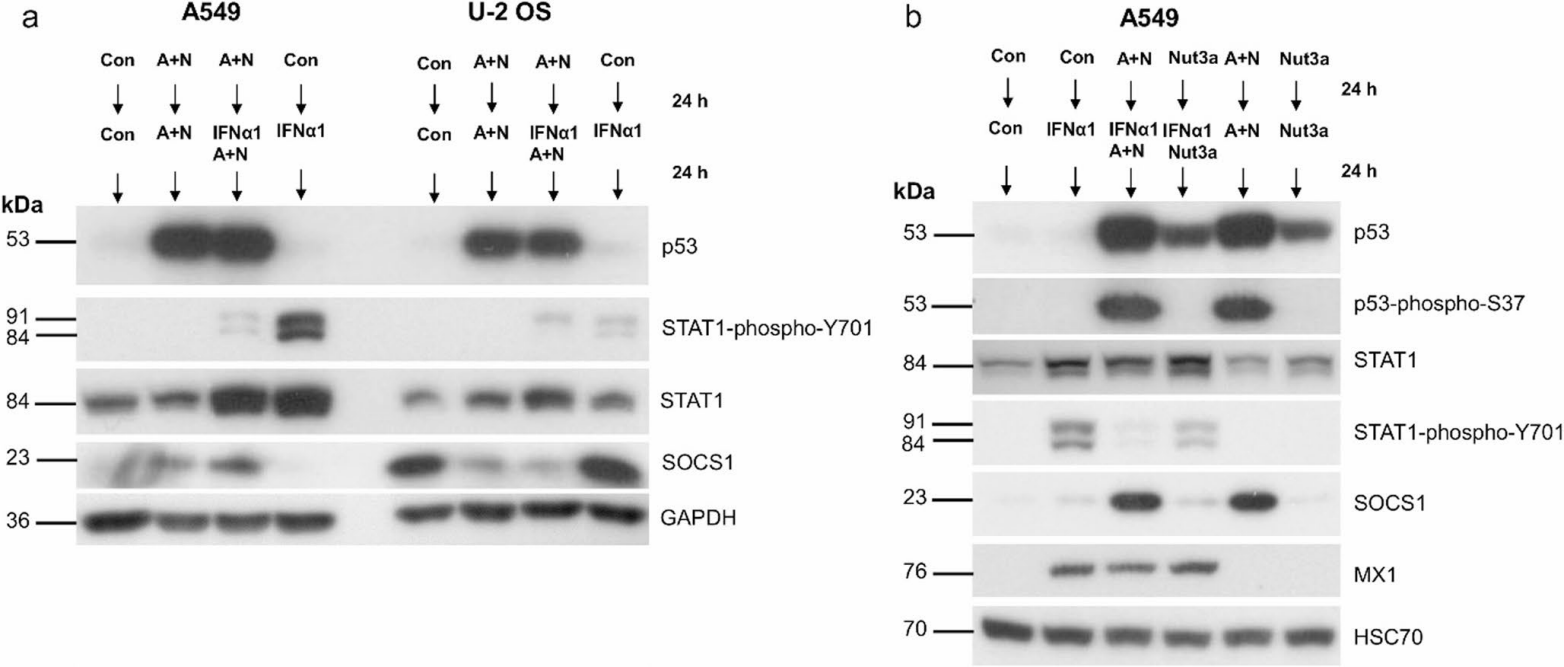
Exposure of cells to actinomycin D and nutlin-3a (A + N) reduces the phosphorylation of STAT1 at Tyr-701 in cells exposed to interferon-α1 (IFNα1). (**a**) The Western blot showing expression of indicated proteins. STAT-phospho-Y701 stands for STAT1 phosphorylated at tyrosine (Y) 701. A549 and U-2 OS cells were pre-exposed to A + N or mock-treated (Con) for 24 h. Subsequently, the treatment with A + N was continued but to one culture plate IFNα1 (1 ng/ml) was added. The cytokine was also added to one plate of control cells. The cells were cultured for additional 24 h. So the total time of treatment with A + N was 48 h and with IFNα1–24 h. (**b**) The experiment performed on A549 cells analogously to the one presented in previous panel. Some cells were exposed to nutlin-3a (Nut3a) instead of A + N combination. For technical reasons GAPDH was used as a loading control in panel a. p53-phospho-S37 stands for p53 with phosphorylated serine 37

To compare how the activation of p53 by A + N modulates the response of cells to interferon-α1, we pretreated A549 or U-2 OS cells with A + N for 24 h (control cells were mock-treated), and subsequently, IFNα1 was added either to cells treated with A + N or to cells growing under control conditions, as shown in Fig. 3a. The extended treatment (up to 48 h) of A549 cells with A + N induces cell cycle arrest at the G1 or G2 phase, but does not results in extensive cell death as shown in our previous experiments [13, 14]. The concentration of cytokine was selected based on the dose-response experiment (supplementary figure S1a). In both cell lines, exposure to IFNα1 resulted in the phosphorylation of STAT1 at Tyr701 (Y701), what is consistent with known molecular consequences of IFNα1 activity. In A549 cells, exposure to A + N strongly attenuated the phosphorylation of STAT1, which was accompanied by the upregulation of SOCS1, whereas in U-2 OS cells, the phosphorylation of STAT1 was not altered significantly even though the expression of SOCS1 significantly decreased. Therefore, in U-2 OS cells, there is no relationship between the expression of SOCS1 and the phosphorylation status of STAT1. Hence, further experiments were performed using A549 cells.

Next, we compared how the strong or weak activation of p53 by A + N or nutlin-3a, respectively, modulates the phosphorylation of STAT1. A549 cells were pre-exposed to either A + N or nutlin-3a, and after 24 h, the cells were treated with IFNα1, as shown in Fig. 3b. The level of p53 activation is visualized by its phosphorylation at serine 37 (S37) [15]. Pre-exposure to A + N attenuated the phosphorylation of STAT1, whereas pre-exposure to nutlin-3a had a weak effect. Consistent with previous findings, SOCS1 was upregulated only in cells exposed to A + N (Fig. 3b). We additionally examined the expression of the MX1 protein, which is encoded by the archetypal IFNα-activated gene [16]. Surprisingly, the level of phosphorylated STAT1 was not associated with the expression of MX1, which was almost identical in all conditions when IFNα1 was present (Fig. 3b). This finding is very odd because phosphorylation of STAT1 is considered a measure of its activation; hence, we expected the lower expression of MX1 in cells pre-exposed to A + N and treated with IFNα1. Thus, even though pretreatment with A + N activates p53 and leads to increased expression of SOCS1, which, in turn, is linked to diminished phosphorylation of STAT1 at Tyr701, it does not impact the expression of the gene (*MX1*) activated by the complex containing STAT1 transcription factor. These findings indicate a serious gap in our understanding of the mechanisms of activation of ISGs.

**Fig. 4:**
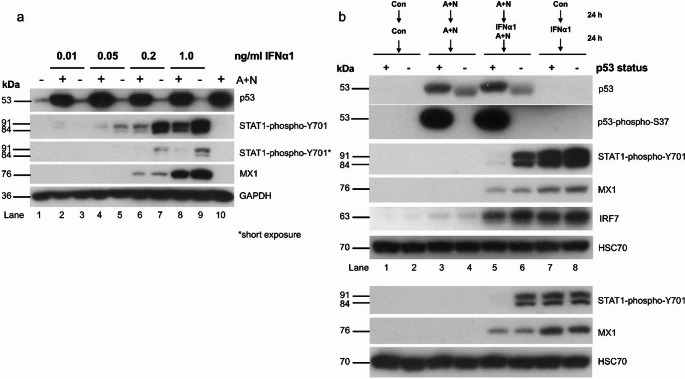
The modulation of STAT1 phosphorylation by A + N does not translate into modulated expression of two selected proteins (MX1, IRF7) coded by the genes activated by IFNα1. (**a**) A549 cells were exposed via the procedure presented in Fig. 3a, but various concentrations of IFNα1 were used. The expression of the indicated proteins was subsequently determined via Western blotting. The expression of STAT1-phospho-Y701 was visualized after long and short (marked by *) exposures. (**b**) The p53-proficient (+) and p53-deficient (-) A549 cells were exposed as indicated (analogously to experiments shown on Fig. 3) and the expression of relevant proteins or their phosphorylated forms (p53 at serine 37 and STAT1 on tyrosine 701) was determined by Western blotting. IFNα1 was used at a concentration of 1 ng/ml. The lower panel shows the results of the independent experiment with 0.5 ng/ml of IFNα1. The lower band visible on the blot probed with anti-p53 antibody represents p53 with deletion at the amino terminus generated by CRISPR/Cas9 technology [14]. The mutation destroys an epitope recognized by antibody against p53 with phosphorylated serine 37

To further explore the relationship between the phosphorylation status of STAT1 and the expression of MX1, we performed a similar experiment (pretreatment with A + N with subsequent treatment with IFNα1); however, we exposed the cells to various concentrations of the cytokine. The results are presented in Fig. 4a. Pre-exposure to A + N reduced the phosphorylation of STAT1 triggered by IFNα1. It was visible at concentrations of IFNα1 ranging from 0.05 to 1.0 ng/ml; however, it did not affect the expression of MX1. A comparison of the amount of phospho-STAT1 between various experimental conditions revealed that despite similar phospho-STAT1 levels, the expression of MX1 clearly differed (lanes 5 versus 6, 7 versus 9 and 7 versus 8). This experiment confirmed the lack of correlation between the amount of phosphorylated STAT1 and the expression of MX1. However, a lower concentration of this cytokine is associated with lower expression of MX1. Thus, even though the activation of p53 is associated with reduced phosphorylation of STAT1, it does not affect the expression of *MX1* gene regulated by phospho-STAT1.

### p53 attenuates the phosphorylation of STAT1 initiated by IFNα1

Exposure of cells to A + N attenuates the phosphorylation of STAT1. Does A + N act in this regard through activation of p53? To answer this question, we tested how p53 status modulates the phosphorylation of STAT1. We employed a mixture of clones of A549 cells with attenuated expression of p53. The p53-deficient cells were prepared as described previously [14]. IFNα1 alone induced strong phosphorylation of STAT1, which was not modulated by p53 status (Fig. 4b, lanes 7 and 8). Preexposure to A + N significantly attenuated the phosphorylation of STAT1 induced by IFNα1 but only in cells with wild-type p53 (Fig. 4b, lanes 5 and 7). As in previous experiments, the phosphorylation of STAT1 was not correlated with the expression of MX1 (compare lanes 5 and 6). Another gene activated by IFNα1 - IRF7 [17], was also unaffected. Thus, the activation of p53 by A + N reduces the phosphorylation of STAT1, but it has unmeasurable influence on the expression of the STAT1 target genes *MX1* or *IRF7*.

### Variable influence of p53 on the expression of genes activated by interferon-α1

**Fig. 5:**
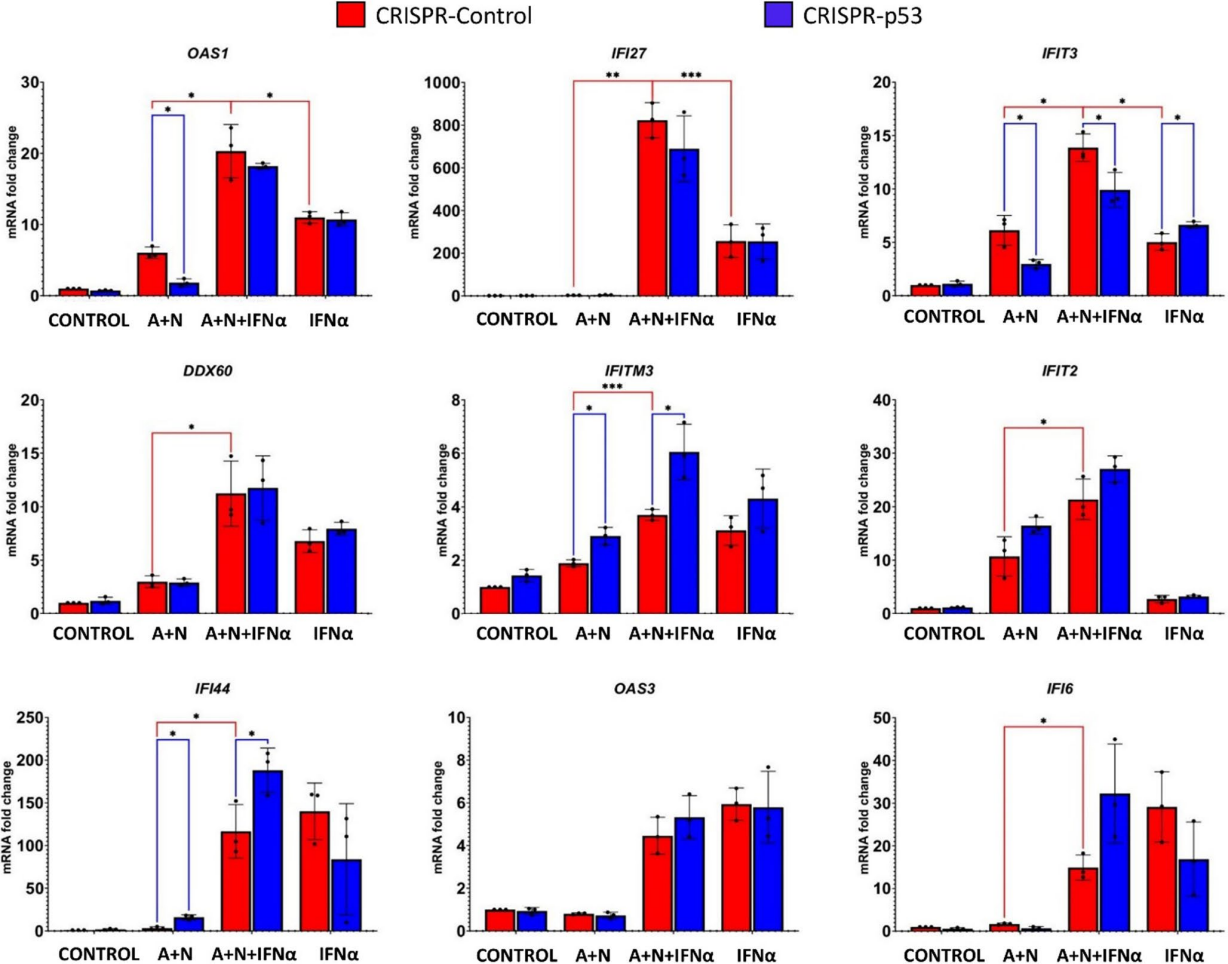
p53 modulates the expression of only a subset of genes activated by IFNα1. p53-proficient (red bars) and p53-deficient (blue bars) A549 cells were exposed to A + N, A + N with IFNα1 or IFNα1 via the two-step procedure presented in Fig. 3a. The IFNα1 concentration was 0.5 ng/ml. The expression of the indicated genes was subsequently determined via semi-quantitative RT‒PCR. Three biological replicates were performed. The *ACTB* gene was used as a reference gene. The influence of p53 status was calculated (blue lines) via an unpaired t test with Welch’s correction, taking into account the false discovery rate (FDR = 10%). Additionally, the statistical significance was calculated for p53-proficient cells (red lines) treated with A + N vs. A + N + IFNα and A + N + IFNα vs. IFNα. For this purpose, calculations were performed via the unpaired t test with Welch’s correction for each group separately (* *p* < *0*.*05*, ** p < 0.*01*, *** p < 0.*001)*

The lack of association between the phosphorylation status of STAT1 and the expression of STAT1 targets, *MX1* and *IRF7*, is surprising. To determine how other genes activated by IFNα1 are modulated by A + N and p53 status, we first treated p53-proficient and p53-deficient cells as presented in Fig. 4b and then we performed gene expression analysis via semi-quantitative RT‒PCR (Fig. 5). In p53-proficient cells, pretreatment with A + N did not reduce the expression of any gene, with the exception of *IFI6* (compare, in p53-proficient cells, the treatment with IFNα1 *versus* treatment with IFNα1 and A + N). Furthermore, in p53-deficient cells, A + N did not reduce expression of *IFI6* triggered by IFNα1. Thus, the expression of *IFI6* was roughly correlated with the phosphorylation status of STAT1.

The p53 status significantly modulated the expression of *IFI44* in cells exposed to A + N or to A + N combined with IFNα1. In p53-deficient cells, this gene shows higher expression. Thus, p53 negatively regulates *IFI44*. Similar expression pattern was found for *IFITM3*, what is consistent with the observations of Wang et al. [18], who demonstrated that p53 negatively regulates this gene.

Both interferon-stimulated genes, *OAS1* and *IFIT3* are upregulated by A + N in p53-dependent fashion (expression significantly lower in p53-deficient cells). Moreover, IFNα1 and A + N collaborate in their activation. In the case of *IFI27* gene, there is very strong collaboration between A + N and IFNα1 in its upregulation but this effect is not regulated by p53. Similar p53-independent cooperation between A + N and IFNα1 in gene activation (although not as spectacular as in the case of *IFI27*) was observed for *DDX60*, and *IFIT2*. The *OAS3* gene was activated only by IFNα1, and neither exposure to A + N nor p53 status modulated its expression. In this context, it is similar to the *MX1* gene.

Based on the semi-quantitative RT‒PCR results, we can conclude that various ISGs respond differently to A + N, IFNα1, and the status of p53. The combination of A + N appears to synergize with IFNα1 in the activation of some genes, but this occurs in a p53-independent manner (e.g., *IFI27* and *DDX60*). p53 promotes the expression of several ISGs including *OAS1* and *IFIT3*. On the other hand, some of the examined ISGs, e.g., *IFITM3* and *IFI44*, are negatively regulated by p53. Thus, under our experimental conditions, the interactions between the signaling systems controlled by p53 and interferon-α1 are complex, even though p53 clearly participates in reducing the phosphorylation of STAT1. Moreover, our treatment modality (A + N) clearly cooperates with IFNα1 in the activation of some genes, but the p53 status has no effect on this interaction. Thus, A + N may be an important regulator of innate immunity via an unknown mechanism that is independent of p53. This is in line with observation that in U-2 OS cells (Figs. 1 and 2), A + N combination can modulate the expression of innate immunity gene (*SOCS1*) regardless of p53 status.

### p53 and IFNγ synergize to activate a subset of interferon-stimulated genes

**Fig. 6:**
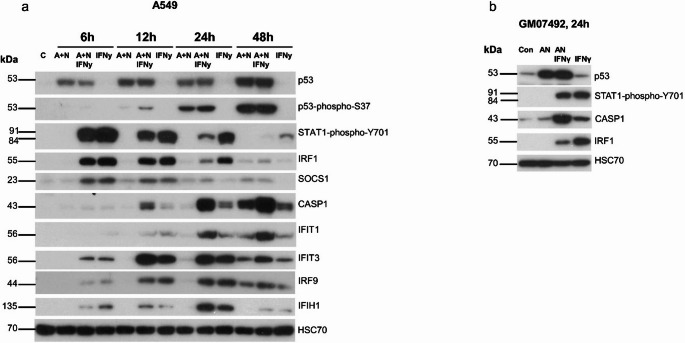
The combination of A + N and interferon-γ (IFNγ) synergize in the activation of a subset of innate immunity genes. (**a**) A549 cells were exposed to A + N, A + N with IFNγ or IFNγ alone for increasing numbers of hours. IFNγ was used at a concentration of 1 ng/ml. The expression of selected proteins was subsequently determined via Western blotting. (**b**) Western blot of lysates from GM07492 normal human fibroblasts exposed to A + N, IFNγ (1 ng/ml) or their combination for 24 h

The STAT1 protein is also phosphorylated in response to other class of interferon, namely interferon-γ. Therefore, SOCS1 activated by p53 may also influence the signaling pathway of this cytokine. Importantly, SOCS1 serves as the primary negative regulator of IFNγ signaling [19]. Hence, we decided to examine how the activation of p53 by A + N impacts the outcome of IFNγ treatment. First, we performed a time-course experiment in which A549 cells were exposed to combination of A + N, IFNγ or A + N combined with IFNγ for 6, 12, 24 and 48 h. By Western blotting, we examined the activation status of p53 (total protein expression and its phosphorylation at serine 37), the phosphorylation of STAT1 (tyrosine 701) and the expression of genes known to be activated by this cytokine (*IRF1*,* IRF9*,* SOCS1*,* CASP1*,* IFIT1*,* IFIT3 and IFIH1*). This Western blot reveals several interesting points (Fig. 6a). The activating phosphorylation of p53 protein increased steadily throughout the entire A + N treatment period, whereas the phosphorylation of STAT1 peaked after 6 h from the start of IFNγ application and then gradually declined. A similar pattern was observed for the expression of SOCS1 and IRF1 in cells exposed to interferon-γ. The expression of CASP1 and IFIT1 increased steadily in cells exposed to A + N or in cells exposed to IFNγ. The expression of IFIH1 peaked after 24 h. When both treatments were combined, the expression of CASP1 and IFIT1 further increased, peaking after 48 h. Interestingly, at the 24 h time point, the phosphorylation of STAT1 and the level of IRF1 were lower in cells co-exposed to A + N and interferon than in cells treated with the cytokine alone. These findings suggest that at some time points, the activation of p53 can reduce the phosphorylation of STAT1. Surprisingly, at 12-hour time point, the level of p53 phosphorylation at Ser37 was higher in cells exposed to A + N and IFNγ then those treated with A + N alone. We measured p53-phospho-S37 expression in several other independent repeats of 12-hour treatment, but the results were not consistent. Definitely, we observe strong collaboration between A + N and IFNγ in upregulation of CASP1, IFIT1 and IFIT3. (Fig. 6a). Moreover, the activation of p53 by combination of A + N with IFNγ resulted in strong upregulation of the CASP1 protein in normal human fibroblasts (Fig. 6b).

**Fig. 7:**
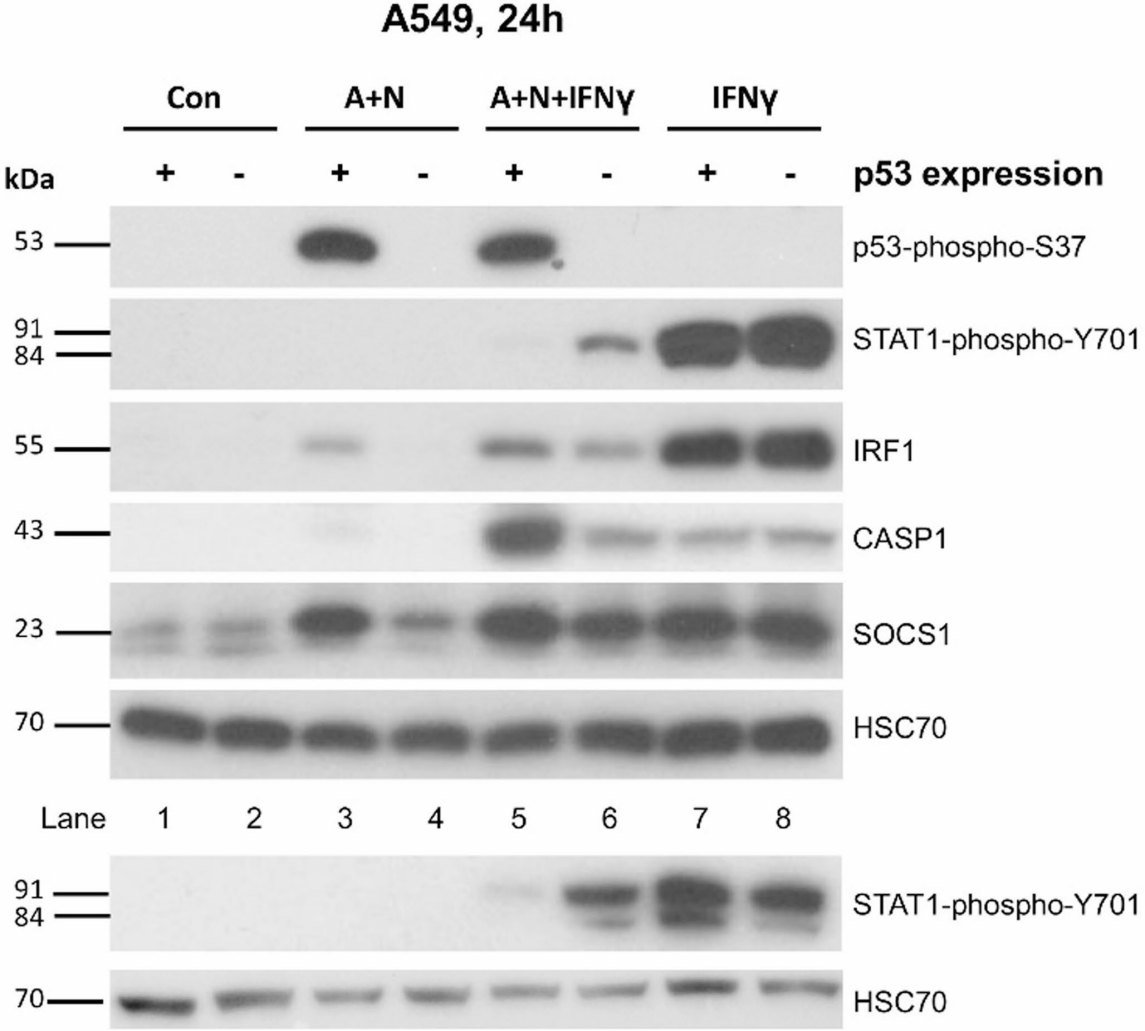
p53 has some effect on reducing the phosphorylation of STAT1 induced by IFNγ. P53-proficient (+) and p53-deficient (-) A549 cells were mock-treated (Con) or exposed either to A + N, or IFNγ or A + N with IFNγ for 24 h. IFNγ was used at a concentration of 1 ng/ml. The expression of the indicated proteins was determined by Western blotting. The lower panel shows the results of an independent experiment

To determine whether p53 impacts on the regulation of STAT1 phosphorylation and the expression of early (*IRF1*,* SOCS1*) and late (*CASP1*) interferon γ-activated genes, we treated p53-proficient and p53-deficient A549 cells, as shown in Fig. 7. This experiment confirms that *SOCS1*, *IRF1* and *CASP1* are regulated by p53 because A + N is unable to upregulate their expression in p53-deficient cells. However, when the cells are additionally exposed to IFNγ, the effect of p53 on the expression of IRF1 and SOCS1 is lost. This is in stark contrast to the expression of CASP1. A + N and IFNγ clearly synergize in the activation of its expression. Moreover, this effect strictly depends on p53 because, in p53-deficient cells, the effect of A + N is absent. Thus, p53 and IFNγ synergize in upregulation of *CASP1* (but not *SOCS1* and *IRF1*) and p53 modulates the IFNγ-induced STAT1 phosphorylation.

**Fig. 8:**
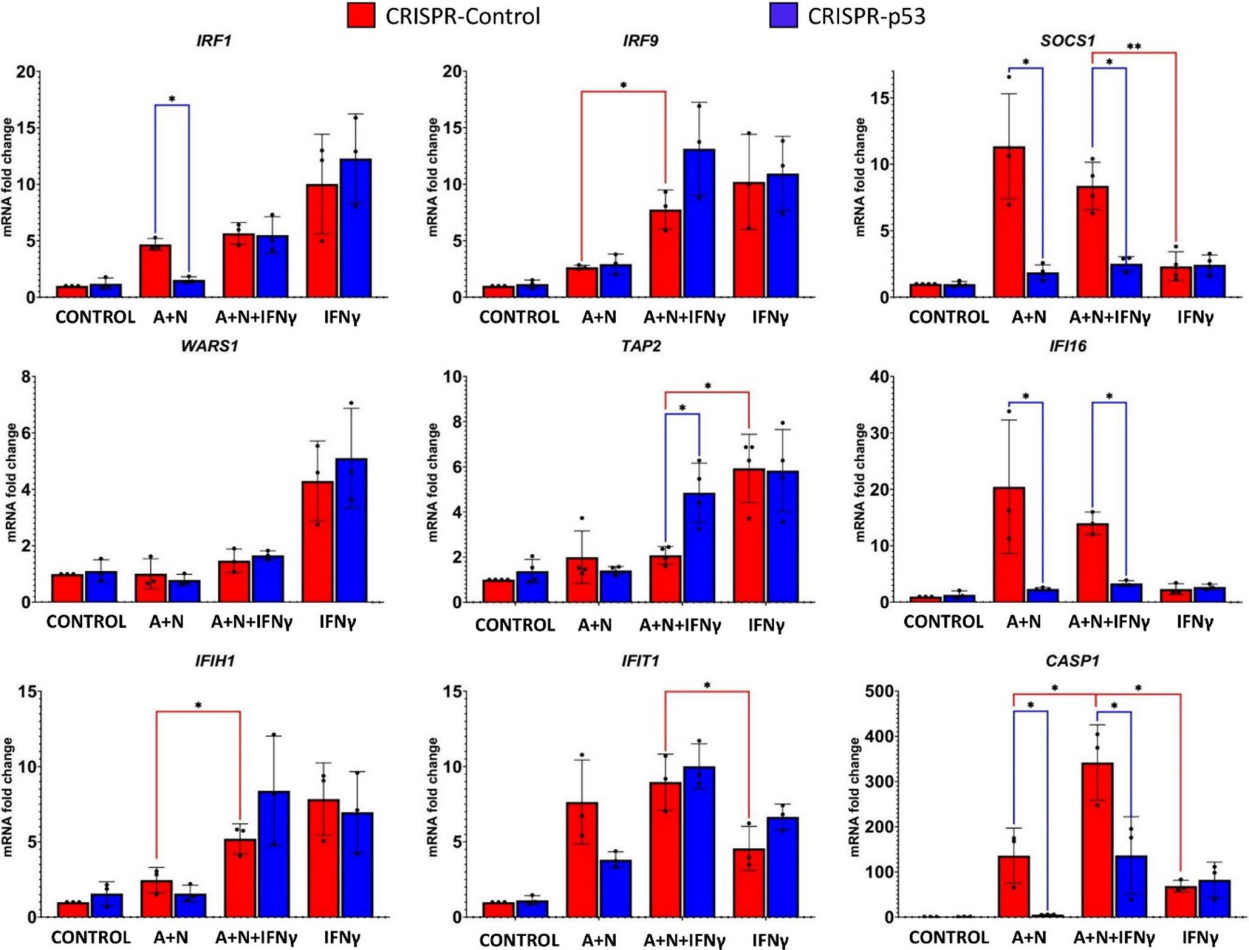
Activated p53 and IFNγ synergize to stimulate the *CASP1* gene, which codes for pro-pyroptotic caspase-1. p53-proficient (red bars) and p53-deficient (blue bars) A549 cells were exposed to A + N, A + N with IFNγ or IFNγ alone for 24 h. Control cells were mock-treated. The IFNγ concentration was 1 ng/ml. The expression of the indicated genes was subsequently determined via semi-quantitative RT‒PCR. Three biological replicates were performed. *ACTB* was used as a reference gene. The influence of p53 status (blue lines) was calculated via an unpaired t test with Welch’s correction, taking into account the false discovery rate (FDR = 10%). Additionally, statistical significance was calculated for p53-proficient cells treated with A + N vs. A + N + IFNγ and A + N + IFNγ vs. IFNγ (red lines). For this purpose, calculations were performed via the unpaired t test with Welch’s correction or the Mann‒Whitney test for each group separately (* *p* < *0*.*05*, ** p < 0.*01)*

To determine how A + N and IFNγ cooperate in the regulation of other genes in p53-proficient and p53-deficient cells, we performed semi-quantitative RT‒PCR analysis on RNA samples isolated from cells exposed as shown in Fig. 7. The results are presented in Fig. 8. We selected genes known to be activated by IFNγ. We did not observe a single pattern of cooperation between A + N and IFNγ. In p53-proficient cells, the expression of only two genes *WARS1* and *TAP2* matched the phosphorylation status of STAT1 (which was lower in A + N + IFNγ than in IFNγ alone),. The expression of *TAP2* was influenced by p53 status. *IFI16* and *SOCS1* show very similar expression patterns under these treatment conditions: they are weakly upregulated by IFNy and strongly activated by A + N, but they are expressed only in p53-proficient cells, which is consistent with observations that they are p53-regulated genes [10, 20]. The expression of *IRF9* and *IFIH1* is governed principally by IFNγ, and A + N does not reduce their expression (this finding is consistent with the Western blotting data; Fig. 6a). The expression of *IRF1* is governed by p53 in cells exposed to A + N, and this combination together with IFNγ does not contribute to its upregulation. In this context, the expression pattern of *CASP1* stands out. First, there is conspicuous synergy between A + N and IFNγ in the activation of this gene. Its upregulation by any treatment is very strong (up to 300-fold). p53 is strictly required for its upregulation by A + N with or without IFNγ. Therefore, semi-quantitative RT‒PCR confirmed the conclusion from the Western blotting data that p53 and IFNγ strongly synergize in the activation of the *CASP1* gene.

**Fig. 9:**
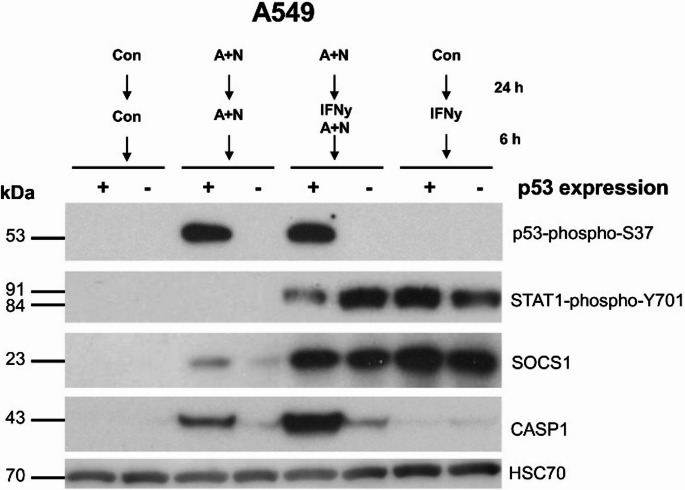
The synergy between activated p53 and IFNγ results in strong upregulation of caspase-1 (CASP1) protein. The p53-proficient (+) and p53-deficient (-) A549 cells were either mock-treated (Con) or exposed to A + N for 24 h. Subsequently, the treatment with A + N and mock-treatment was continued but some cells were additionally exposed to 1 ng/ml IFNγ for the next 6 h as shown on the picture

Because p53 is progressively activated during treatment with A + N while the phosphorylation of STAT1 and the expression of some genes activated by IFNγ are the strongest after 6 h of cytokine treatment (Fig. 6a), the next experiment had a different setup. First, we exposed the cells for 24 h to A + N and then exposed them for 6 h to IFNγ, as demonstrated in Fig. 9. Western blot revealed that in p53-proficient cells, treatment with A + N slightly reduced the phosphorylation of STAT1. SOCS1 is activated in a p53-dependent manner only in cells exposed to A + N. The activation of this gene is stronger after 6 h of exposure to IFNγ than after 30 h of exposure to A + N. Thus, under these experimental conditions, IFNγ is a much stronger activator of SOCS1 than p53 is. A similar conclusion can be drawn from Fig. 6. Consistent with the results of previous experiments, this blot revealed very strong synergy between p53 and IFN-γ in the activation of CASP1.

**Fig. 10:**
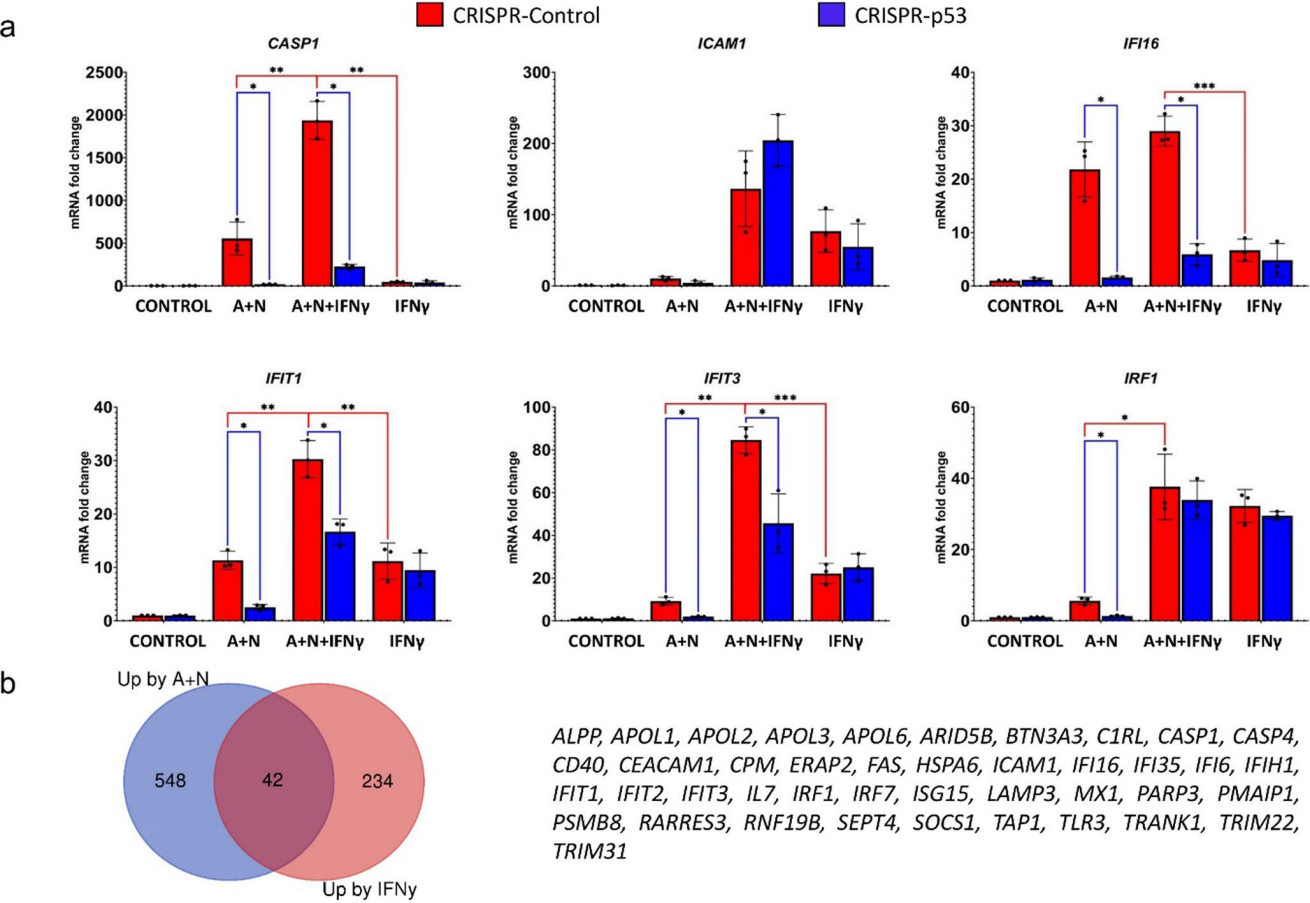
Activated p53 and IFNγ synergize in the stimulation of a subset of innate immunity genes. (**a**) p53-proficient (red bars) and p53-deficient (blue bars) A549 cells were exposed to A + N, A + N with IFNγ or IFNγ alone as described in Fig. 9 (treatment mode 24 h + 6 h). The control cells were mock-treated. The IFNγ concentration was 1 ng/ml. The expression of the indicated genes was subsequently determined via semi-quantitative RT‒PCR. Three biological replicates were performed. *GAPDH* was used as a reference gene. The influence of p53 status was calculated via an unpaired t test with Welch’s correction (blue lines), taking into account the false discovery rate (FDR = 10%). Additionally, statistical significance was calculated for p53-proficient cells treated with A + N vs. A + N + IFNγ and A + N + IFNγ vs. IFNγ (red lines). For this purpose, calculations were performed via the unpaired t test with Welch’s correction for each group separately (* *p* < *0*,*05*, **p < 0,*01*, *** p < 0,*001* (**b**) Venn diagram showing the number of common genes upregulated by A + N or by IFNγ in A549 cells. The genes upregulated at least 4-fold by A + N (30-hour treatment) were taken from our previous paper [6]. *CASP1*,* IL7* and *IRF1* were also included because they were found to be strongly activated by A + N in our earlier projects [10, 14]. The genes upregulated at least 2-fold by IFNγ after 24-hour treatment were from the work of Sanda et al. [22] and the data were reached via Interferome database [21]. The common 41 genes activated by A + N and by IFNγ are listed on the right

Next, we performed semi-quantitative RT‒PCR on RNA samples isolated from cells treated as shown in Fig. 9. The results are presented in Fig. 10a. Under these experimental conditions, *IRF1* is more potently activated by IFNγ than by p53, and these two molecules do not cooperate in its activation. On the other hand, the *IFI16* gene is more potently activated by p53 than by IFNγ, and these two molecules appear to cooperate in its expression. A + N and IFNγ also cooperate in the activation of *ICAM1*, but p53 does not seem to participate in this process. Finally, we observed very strong and synergistic cooperation between p53 and IFNγ in the activation of *CASP1*, *IFIT1* and *IFIT3*, the synergy being the strongest in the case of the *CASP1* gene. Thus, even though the treatment with A + N decreases the phosphorylation of STAT1 initiated by IFNγ, A + N does not reduce the expression of most examined genes activated by this cytokine. In the contrary, A + N cooperates with IFNγ in activating a subset of genes, particularly those that are upregulated later during exposure to this cytokine.

To compare the sets of genes activated by A + N or by IFNγ, we searched the Interferome, an open access database of types I, II and III interferon-regulated genes [21]. In this database, we found a study on genes upregulated by IFNγ in A549 cell line [22]. We compared the list of genes activated at least 4-fold by A + N in A549 cells [6] with the list of genes upregulated at least 2-fold by IFNγ. We lowered the limit for IFNγ to have comparable number of genes in both groups. The results are presented on Fig. 10b. There are 42 genes, which can be upregulated by both A + N (most likely via p53) and by IFNγ in the same cells. Thus, 15% of genes upregulated by this cytokine can also by activated by A + N. These common genes are presented on Fig. 10b. This extensive list of immunity genes (activated by IFNγ), which are also positively regulated by p53 further supports the model, according to which p53 is important regulator of immunity [23]. The listed genes apparently have very important functions because they are activated by two major defense systems governed by p53 and IFNγ.

### IFNγ sensitizes cells to FAS ligand but does not synergize with p53 in the induction of apoptosis

**Fig. 11:**
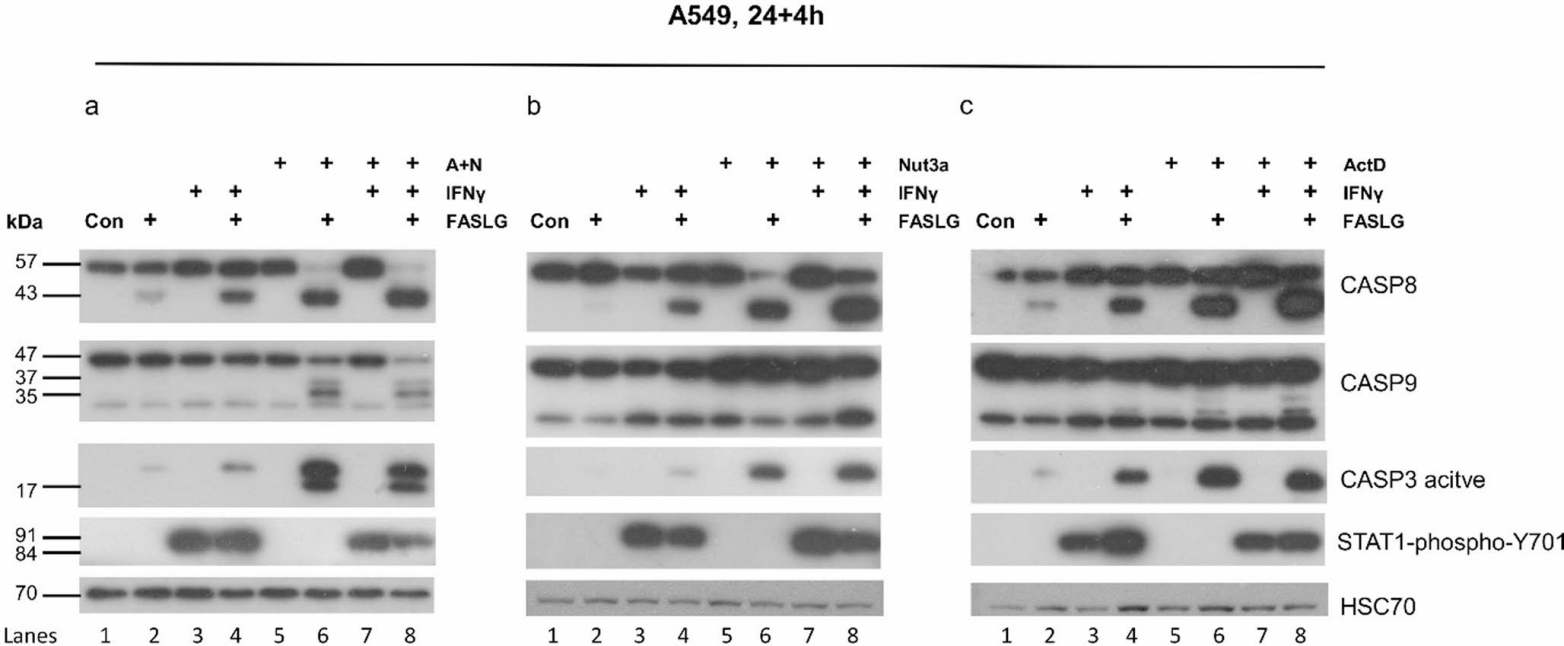
Interferon-γ sensitizes cells to the apoptosis induced by FAS ligand. A549 cells were exposed for 24 h to IFNγ (2 ng/ml), A + N, Nutlin-3a (Nut3a), actinomycin D (ActD) or their combination as indicated. The medium was subsequently changed, and the cells were exposed to FASLG (20 ng/ml) for 4 h. The expression of the indicated proteins was determined by Western blotting. The full-length forms of caspases-8 and 9 have size of 57 kDa and 47 kDa, respectively. The size of cleaved caspase-8 is 43 kDa, and of cleaved caspase-9 is 35 kDa and 37 kDa. A band below 35 kDa is apparently an unspecific protein detected by the anti-caspase-9 antibody

The p53 protein activated by A + N synergizes with IFNγ in activation of a subset of genes regulated by both proteins. This synergy was the most apparent for the *CASP1* gene, which is known for its ability to induce proinflammatory death called pyroptosis [24], but it can also induce apoptosis [25]. Recently, we demonstrated that p53 activated by A + N strongly sensitizes cells to the apoptosis induced by the ligand (FASLG) of the death receptor FAS [26]. IFNγ can also sensitize various cells to FAS-induced apoptosis [27, 28]. Interestingly, one study showed that caspase-1 mediates FAS-induced cell death in astrocytoma cells [29]. Hence, we investigated whether these two factors (activated p53 and IFNγ) cooperate in the sensitization of cells to apoptosis triggered by FASLG. The cells pre-exposed to A + N and treated with FASLG show typical morphology of apoptotic cells (supplementary figure S3). We exposed the cells as shown in Fig. 11a. Consistent with the observations of others, pre-exposure of cells to IFNγ sensitized cells to the proapoptotic activity of FASLG. The cells exhibited stronger signal from cleaved caspase-8, which is triggered by the active FAS receptor (FASR), and more active caspase-3, which is the executioner caspase (compare lanes 2 and 4 in Fig. 11a, b and c). In line with our previous findings [26], pretreatment of cells with A + N also resulted in the appearance of more cleaved caspase-8 and more active caspase-3 than did treatment with the FAS ligand alone (compare lanes 2 and 6). In cells treated with combination of A + N + FASLG, we also observed cleaved caspase-9, what suggested that the proapoptotic effect of A + N + FASLG was stronger than that of IFNγ + FASLG. However, when the pretreatment with A + N was combined with the pretreatment with IFNγ, there was no increase in the amount of cleaved caspase-8, −9 or −3 (lanes 6 versus 8). Thus, even though IFNγ or A + N individually sensitize cells to apoptosis triggered by FASLG, they apparently do not cooperate in this regard, at least in a way that could be measured by our test.

The effect of p53 activated by A + N could overpower the relatively small influence of IFNγ on the apoptosis induced by FAS ligand, hence, in the next step, we employed more modest activation of p53 either by nutlin-3a (Fig. 11b) or by actinomycin D (Fig. 11c). In the treatment modality presented on Fig. 11b and c, the proapoptotic effect of FAS ligand was relatively weak because caspase-9 was not (nutlin-3a) or was barely (actinomycin D) activated (compare lanes 6 and 8 from panel a with the lanes 6 and 8 from panels b and c). In these two experiments, IFNγ also distinctly sensitized the cells to the proapoptotic activity of FASLG. However, IFNγ and p53 do not measurably cooperate in the sensitization of cells to the proapoptotic activity of FASLG (the signals from cleaved caspase-8 and activated caspase-3 are very similar in lanes 6 and 8).

**Fig. 12:**
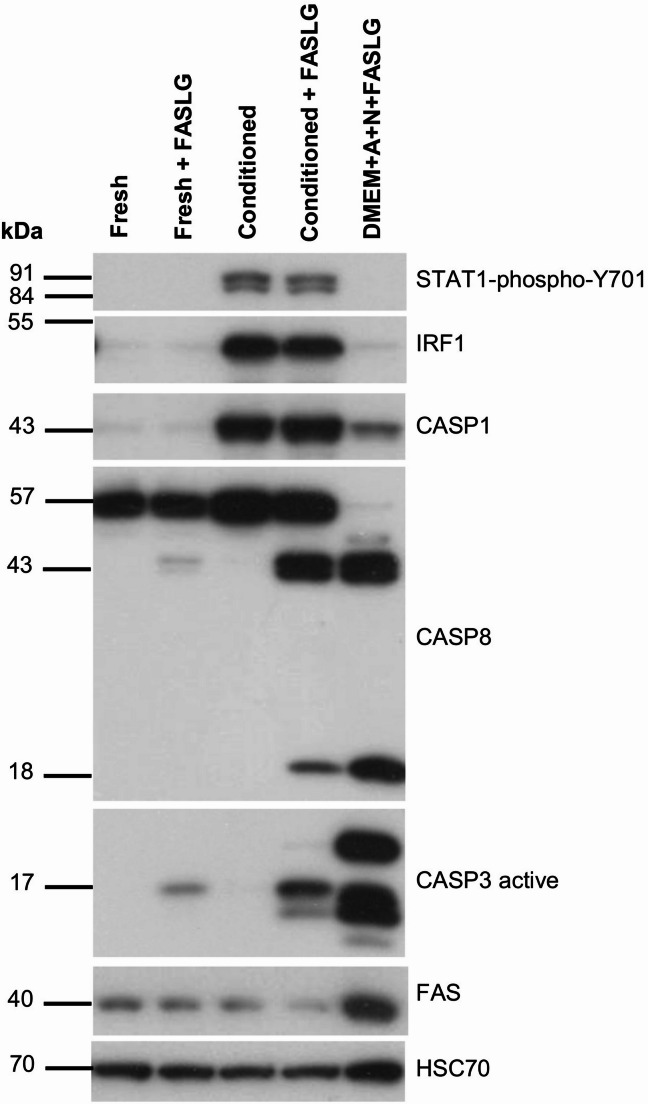
Conditioned medium from NK-92 cells sensitizes A549 cancer cells to the proapoptotic activity of FAS ligand. Conditioned RPMI-1640 medium was prepared by growing NK-92 cells at concentration of two hundred thousand cells per 1 ml for 24 h. After centrifugation to remove cells, the medium was added to the culture of A549 cells for 24 h. After that time FASLG was added (50 ng/ml) to one plate of cells for 4.5 h. The negative controls were A549 cells growing for 24 h in fresh RPMI-1640. After 24 h in fresh medium, the FASLG was added to one plate of cells. As a positive control (strong induction of apoptosis), A549 cells were incubated in DMEM medium with A + N for 24 h to activate p53. The medium was subsequently changed, and 50 ng/ml FASLG was added for 4.5 h. The expression of indicated proteins was determined by Western blotting. The full length caspase-8 is 57 kDa and its cleaved forms are at 41/43 kDa (partially cleaved) and 18 kDa (totally cleaved). Active caspase-3 migrates as two major bands – 17 kDa and 19 kDa. FAS is the receptor for FASLG. As a product of p53-activated gene FAS is upregulated by treatment with A + N

The ability of IFNγ to sensitize cells to FAS-induced apoptosis has been reported by others [27, 28] and confirmed by our experiments. These studies employed purified IFNγ. We decided to determine whether natural sources containing this cytokine can elicit similar effects. To this end, we employed conditioned medium from the NK-92 cell line, which has the phenotype of natural killer cells [30]. This type of innate immune cells, when activated, secretes this cytokine [31]. NK-92 cells are widely used as surrogate NK cells in basic research; they can also serve as an experimental anticancer therapy [30], and they secrete IFNγ [32]. In A549 cancer cells, the conditioned medium from NK-92 cells caused the phosphorylation of STAT1 at Tyr701, as well as the upregulation of the IRF1 and CASP1 proteins, which is consistent with the activity of IFNγ (Fig. 12). We found that this conditioned medium distinctly sensitized A549 cells to the apoptosis triggered by FASLG, which was visible as the appearance of cleaved, active forms of caspases − 8 and − 3. However, the effect of conditioned medium on the sensitization to apoptosis triggered by FASLG was smaller than the effect of p53 activated by A + N. These results show that NK-92 cells can induce cell death not only directly by forming immunological synapses but also indirectly through the secretion of cytokines, which sensitize target cells to the apoptosis triggered by FASLG.

## Discussion

The interactions between interferon and p53 signaling pathways were presented in our recent review [26] and in reviews published by others [2, 33]. Interferons belong to distinct protein families (types) signaling through different receptors. We studied type I interferon-α1 and type II interferon-γ, which are negatively regulated by SOCS1. Previously, we found that the *SOCS1* gene is activated by p53 in the A549 non-small cell lung cancer cell line (*TP53* wild-type) [10], what suggested that p53 can negatively regulate interferon signaling. In current study, we confirmed and extended this observation by showing that *SOCS1* is also induced by p53 activators in NCI-H292 cell line (*TP53* wild-type). To our surprise, we found that, in the U-2 OS cells (*TP53* wild-type), SOCS1 was repressed by p53 activated by nutlin-3a but not by p53 activated by A + N. This observation is very interesting, because it raises very important question - why is p53 able to suppress SOCS1 when activated by nutlin-3a and is not able to do so when activated by A + N? This finding supports the idea that p53 activated by various stress factors has different biological properties. Moreover, the direction of *SOCS1* gene regulation by p53 depends on the cell type, what is consistent with published transcriptomic data, which revealed the upregulation of *SOCS1* by p53 in 12 reports and its downregulation by p53 in 9 reports [4]. Our data also support the observations that cancer cells vary in the steady-state level of SOCS1 [34]. This duality of *SOCS1* is also manifested in its behavior either as an oncogene or as a tumor suppressor depending on cellular context [35].

P53 activated by A + N attenuates the phosphorylation of STAT1 at Tyr701 induced by interferon-α1. Thus, p53 has the potential to attenuate the expression of ISGs. However, surprisingly, attenuated phosphorylation of STAT1 did not translate into attenuated expression of tested genes stimulated by IFNα1*—MX1* and *IRF7*. To study the expression of more ISGs, we performed semi-quantitative RT‒PCR analysis and observed a highly variable pattern of gene regulation. The activation of three genes (*IFI6*, *IFI44*, and *IFITM3*) was attenuated by p53. The synergy between A + N and IFNα1 was very strong in case of activation of *IFI27*, however, it was not influenced by p53. The IFI27 protein may be involved in the regulation of cell sensitivity to apoptosis [36]. And finally, IFNα1 and p53 cooperated in activation of *IFIT3*. This experiment led to several conclusions. First, p53 is able to attenuate the expression of a subset of genes activated by IFNα1, which is consistent with the ability of p53 to attenuate the phosphorylation of STAT1. Second, p53 activated by A + N can collaborate with IFNα1 in the upregulation of a subset of genes, e.g. *IFIT3*. Finally, the regulation of *IFI27* points toward the existence of a p53-independent mechanism that is able to activate innate immunity genes, which is triggered by actinomycin D and nutlin-3a.

The ability of p53 to attenuate the expression of a subset of genes stimulated by IFNα was observed by Wang et al. [18], who reported that p53 negatively regulates the expression of interferon-induced transmembrane proteins, such as IFITM1, IFITM2 and IFITM3. In our experiment *IFITM3* was also repressed by p53 (Fig. 5). Wang et al. [18] reported that the activation of p53 leading to the repression of *IFITM* genes facilitated the infectivity of influenza A virus. On the other hand, Muñoz-Fontela et al. [37] reported that infected mouse and human cells with functional p53 displayed markedly decreased viral replication early after infection. This inhibition of viral replication was mediated by a p53-dependent increase in interferon signaling, what is consistent with our observation that IFNα1 and p53 collaborate in the activation of *IFIT3* (Fig. 5). These apparent discrepancies pose serious biological questions. Why does p53, which stimulates the expression of the *STING1* gene [10], a stimulator of interferon genes (hence the name), attenuate the expression of some genes induced by this cytokine? We believe that this system is highly complex because it is the result of a prolonged arms race between viruses and cells. Consequently, a measure “devised” by one system (e.g., a virus) is countered by a corresponding countermeasure “devised” by the other system (e.g., a cell). We observe an evolutionary snapshot of the current state of the battle. It is also possible that inhibition of STAT1 phosphorylation by p53 is an element of the negative-feedback loop of this signaling system. Our experiment (Fig. 5) also revealed that p53 and interferon-α1 activate common genes, e.g., *IFIT3* and *OAS1*. Thus, p53 can attenuate the expression of some genes activated by IFNα1 (e.g., *IFITM3*,* IFI44*), and it can activate others (*OAS1*,* IFIT3*). These findings indicate that each ISG has its own program of regulation when interferon is combined with the activation of p53.

Next, we studied how p53 regulates the expression of genes activated by IFNγ belonging to a separate type of interferon than IFNα1. The time course experiment revealed that after 24 h of incubation, the activation of p53 was associated with attenuated phosphorylation of STAT1 (Fig. 6). Moreover, p53 contributes to the repression of STAT1 phosphorylation in cells exposed to IFNγ (Fig. 7). Reduced phosphorylation of STAT1 was associated with the reduced expression of *IRF1*, which is a gene activated early during IFNγ exposure (reviewed by Castro et al. [31]). This correlation was not detected in the case of another early gene, *SOCS1* (Fig. 7). When the expression of other interferon targets was examined, the picture became more complicated. Consistent with observations by others [38], some genes are activated by IFNγ early during exposure, and some genes are activated late (Fig. 6). *IRF1* and *SOCS1* are early genes, whereas *CASP1*, *IFIT1* and *IFIT3* are late genes (Fig. 6). The late gene *CASP1* is activated by the product of the early gene *IRF1*, which encodes a transcription factor [39]. Co-treatment with IFNγ and A + N synergistically induced the expression of three late genes, *CASP1*,* IFIT1* and *IFIT3* (Figs. 6, 8 and 10). The strongest synergy was observed for the *CASP1* gene. This is an original and very important observation because it indicates that these two signaling systems strongly promote the cellular functions executed by this caspase. The best studied function of CASP1 is the induction of pyroptosis, the regulated, pro-inflammatory form of death [24], however, caspase-1 can also induce apoptosis triggered by IFNγ [39]. The molecular mechanism of the synergy between IFNγ and p53 in the activation of CASP1 remains unclear. The late genes activated by IFNγ are not induced by the dimer of phosphorylated STAT1, but by the product of the early gene *IRF1*, which is a transcription factor [31]. Intriguingly, when CASP1 becomes strongly upregulated by IFNγ (24–48 h), the expression of IRF1 returns to the baseline level (Fig. 6a, supplementary Figure S2). Thus, this system may be more complex, with the involvement of additional gene regulatory proteins.

The effect of synergy between p53 and IFNγ was even better visible when we employed a different treatment strategy, namely, we first treated the cells with A + N for 24 h (to upregulate the p53 target genes) and then we exposed them to IFNγ for 6 h. The semi-quantitative RT‒PCR in this case also revealed very strong co-operation of p53 and IFNγ in the upregulation of *CASP1*, *IFIT1*,* IFIT3* and *ICAM1*, another target of IFNγ (Fig. 10). In the case of the *IFI16* gene, which is positively regulated by p53 [20] and IFNγ [40], both proteins act additively in its activation. Thus, different genes regulated by IFNγ and p53 respond differently to simultaneous activation of both signaling systems. Our findings also support the notion that interferon-γ and p53 have common target genes, which code for proteins that participate in the same biological process, e.g., antigen presentation [41, 42], sensitization of cells to apoptosis induced by FASLG [26–28, 43], inhibition of the cell cycle [5, 44] and inflammasome formation [45].

Although IFNγ and p53 sensitize cancer cells to the proapoptotic activity of FASLG, we did not observe any collaboration between these two factors in the induction of apoptosis (Fig. 11). This finding does not rule out collaboration. However, it suggests that our experimental conditions, show no apparent synergy between p53 and IFNγ in the induction of apoptosis triggered by death receptors.

To the best of our knowledge, we noticed for the first time that conditioned medium from NK-92 cells, which contains IFNγ [32], can sensitize cancer cells to apoptosis triggered by FASLG. The conditioned medium induces phosphorylation of STAT1 and activation of early (*IRF1*) and late (*CASP1*) genes stimulated by IFNγ. The observation that the factors secreted by NK-92 cells (mostly IFNγ but possibly other cytokines may have also participated) sensitize cancer cells to apoptosis induced by FAS ligand suggests the potential therapeutic strategy. Specifically, combining NK-92 cell infusion with agonists of FAS or other death receptors could offer promising therapies.

## Materials and methods

### Cell culture and treatment

A549 (RRID: CVCL_0023, lung adenocarcinoma), NCI-H292 (RRID: CVCL_0455, mucoepidermoid pulmonary carcinoma), NCI-H1299 (RRID: CVCL_0060, large cell carcinoma of the lung) and U-2 OS (RRID: CVCL_0042, osteosarcoma) cell lines from ATCC (Manassas, VA, USA) were cultured in low-glucose DMEM supplemented with 10% fetal bovine serum (FBS; Invitrogen, Carlsbad, CA, USA). NK-92 cells (RRID: CVCL_2142, malignant non-Hodgkin lymphoma) from ATCC were cultured on RPMI-1640 medium supplemented with 2 mM glutamine, 1 mM sodium pyruvate, 20 ng/ml interleukin-2 (Miltenyi Biotech, Bergisch Gladbach, Germany), 12.5% FBS, and 12.5% horse serum (Biowest, *Nuaillé*, France). GM07492 normal human fibroblasts (Coriell Cell Repositories, Camden, NJ) were cultured in low-glucose DMEM supplemented with 15% FBS. All media were supplemented with penicillin/streptomycin solution. The cells were incubated at 37 °C and 5% CO_2_ with saturated humidity.

Stock solutions of the following chemicals were prepared in DMSO (dimethyl sulfoxide): actinomycin D (10 µM; Sigma‒Aldrich, St. Louis, MO, USA), camptothecin (10 mM; Calbiochem-Merck, Darmstadt, Germany), and nutlin-3a (10 mM; Selleck Chemicals LLC, Houston, TX, USA). The stock solutions were diluted in culture medium to the following concentrations: 5 nM actinomycin D, 5 µM nutlin-3a, and 5 µM camptothecin. The control cells were mock-treated with medium containing DMSO. Interferons -α1 and -γ were purchased from Cell Signaling Technology (Danvers, MA, USA). The stock solutions were prepared in PBS at 75 µg/ml (IFNα1) or sterile water at 100 µg/ml (IFNγ). The range of interferons concentration was chosen based on dose-response experiments presented in the supplement (Fig. S1). The final concentrations are given in the Results section. FASLG was prepared in sterile water (50 µg/ml; ACRO Biosystems).

### Generation of p53-deficient cells

The generation of p53-deficient A549 and U-2 OS cells via CRISPR/Cas9 technology and the selection of p53 knockout clones from U-2 OS cells were described previously [14].

### Western blotting

The preparation of whole-cell lysates via IP buffer supplemented with protease and phosphatase inhibitors was described previously [10]. Aliquots of lysates (35–50 µg) were separated by SDS‒PAGE on 8% or 13% gels and electrotransferred onto PVDF membranes. Before incubation with primary antibodies, the membranes were incubated for 1 h at room temperature in blocking solution (5% skim milk in PBS with 0.1% Tween-20). Anti-phospho-Ser37-p53, anti-phospho-Tyr701-STAT1 (D4A7), anti-STAT1, anti-MX1, anti-CASP3 (5A1E), anti-CASP8 (1C12), anti-CASP9, anti-FAS (FAS receptor), anti-IFIH1 (MDA5), anti-IRF1 and anti-IFIT1 (D2X9Z) antibodies were obtained from Cell Signaling Technology (Danvers, MA, USA). Anti-p53 (DO-1) and loading control anti-HSC70 (B-6) antibodies were obtained from Santa Cruz Biotechnology (Dallas, TX, USA). The anti-SOCS1 antibody (clone 4H1) was from EMD Millipore (Temecula, CA, USA). Anti-CASP1 (ab179515) and anti-IFIT3 (ab95989) antibodies were obtained from Abcam (Cambridge, UK). The anti-GAPDH loading control antibody was from Merck (Sigma) (Darmstadt, Germany). Anti-IRF7 and anti-IRF9 antibodies were obtained from Proteintech (Rosemont, IL). All incubations with primary antibodies were performed overnight at 4 °C in blocking solution. HRP-conjugated secondary antibodies (anti-mouse, anti-rabbit) were detected via chemiluminescence (SuperSignal West Pico or SuperSignal West Femto Chemiluminescent substrate (Thermo Fisher Scientific, Waltham, MA, USA)).

Unless stated otherwise, the experiments for Western blots were performed once. The key findings were corroborated in similar experiments which followed or were tested using alternative method, e.g. semi-quantitative RT-PCR.

### Gene expression analysis by semi-quantitative RT-PCR

After treatment, the cells were harvested via trypsinization, washed with PBS, frozen on dry ice and stored at −80 °C. Total RNA samples were isolated via an RNeasy mini kit (Qiagen, Hilden, Germany). cDNA was synthesized with MuLV reverse transcriptase and random hexamers (Applied Biosystems, Foster City, CA, USA). Gene expression was measured via Real-Time 2x PCR Master Mix SYBR (A&A Biotechnology, Gdynia, Poland). The sequences of the primers used for semi-quantitative RT‒PCR are given in supplementary Table S1. Amplification was performed on a CFX96 Real-Time System (Bio-Rad, Hercules, CA, USA). For each semi-quantitative RT‒PCR run, cDNA samples were amplified in triplicate. Relative quantitation of mRNA was carried out via the ΔΔCT method, with *ACTB* or *GAPDH* used as a reference. The means and standard deviations were calculated from three biological replicates.

### Preparation of conditioned medium from NK-92 cells for incubation with cancer cells

Two hundred thousand NK-92 cells per ml were incubated in their growth medium for 24 h. Subsequently, the cell suspension was centrifuged to bring the cells to the bottom of the tube, and the cleared medium was added to the culture of A549 cells for 24 h. The negative control A549 cells were incubated in fresh medium for the NK-92 cells. As a positive control, A549 cells were incubated in DMEM with A + N for 24 h to activate p53. The medium was subsequently changed, and 50 ng/ml FASLG was added for 4.5 h, as indicated in Fig. 12. The cells were harvested via trypsinization.

### Statistical analysis

All tests were performed via GraphPad Prism version 10.2.2 for Windows (GraphPad Software, Boston, Massachusetts, USA; www.graphpad.com.) The types of tests are mentioned in the relevant figure captions.

## Electronic supplementary material

Below is the link to the electronic supplementary material.

Supplementary Material 1

Supplementary Material 2

## Data Availability

All the data are contained within the manuscript; all the raw data from our study are available from the corresponding author upon reasonable request.
