# Supplementary material for: The puzzling regulation of the interferon signaling system by the p53 tumor suppressor protein
Source: Cell Mol Life Sci. 2025 Jun 13;82(1):233. doi: 10.1007/s00018-025-05763-0 (PMC12165926; doi:10.1007/s00018-025-05763-0)
Supplement: Supplementary file 1 — Supplementary Material 1 [file 18_2025_5763_MOESM1_ESM.docx]

**Table S 1. The sequence of primers used for semi-quantitative RT-PCR**

| **Gene name** | **Direction** | **Sequence 5’🡪3’** | **The size of PCR product** | **Annealing temperature [^o^C]** |
| --- | --- | --- | --- | --- |
| ***ACP5***  *(Acid Phosphatase 5)* | Forward | CTTTGTAGCCGTGGGTGACT | 103 | 69 |
|  | Reverse | CAGGATCTGCACAGTCCGAG |  |  |
| ***ACTB***  *(Actin, Beta)* | Forward | CAAATAAAGCCATGCCAATC | 144 | 56 |
|  | Reverse | GCAAGCAGGAGTATGACGAG |  |  |
| ***CASP-1***  *(Caspase-1)* | Forward | TCGCTTTCTGCTCTTCCACA | 118 | 68 |
|  | Reverse | TCCACATCACAGGAACAGGC |  |  |
| ***DDX60***  *(DEAD-Box Helicase 60)* | Forward | GCAACCCAGAGTCATGGACA | 114 | 54 |
|  | Reverse | ACAAGTCCAGCAAACCCCAT |  |  |
| ***GAPDH***  *(Glyceraldehyde 3-Phosphate Dehydrogenase)* | Forward | TTCCATGGCACCGTCAAGGC | 173 | 63 |
|  | Reverse | TGCAAATGAGCCCCAGCCTTCT |  |  |
| ***ICAM1***  *(Intercellular Adhesion Molecule 1)* | Forward | AACCTGCCTTTCCCCAGAAG | 190 | 67 |
|  | Reverse | ACCGCTGAGTGTCATTGTGA |  |  |
| ***IFI6***  *(Interferon Alpha Inducible Protein 6)* | Forward | AATGCGGGTAAGGATGCAGG | 200 | 67 |
|  | Reverse | CCATTCAGGATCGCAGACCA |  |  |
| ***IFI16***  *(Interferon Alpha Inducible Protein 16)* | Forward | GAGCAAGCCAGCACTAGTCA | 119 | 56 |
|  | Reverse | CGGAACCGCAGGATGTTGTA |  |  |
| ***IFI27***  *(Interferon Alpha Inducible Protein 27)* | Forward | CTTCACTGCGGCGGGAATC | 158 | 59 |
|  | Reverse | CCAGGATGAACTTGGTCAATCC |  |  |
| ***IFI44***  *(Interferon Alpha Inducible Protein 44)* | Forward | ACGAATTCTGCTGCTGGGTC | 100 | 52 |
|  | Reverse | CACCAAAGCCTGATGCGTTAC |  |  |
| ***IFIH1***  *(Interferon Induced With Helicase C Domain 1)* | Forward | CTGCAAAAGAAGGAAATCGCA | 102 | 60 |
|  | Reverse | ACGCATCTATCATTCGAATTGTGT |  |  |
| ***IFIT1***  *(Interferon Induced Protein With Tetratricopeptide Repeats 1)* | Forward | TGGCAGAAGCCCAGACTTAC | 187 | 66 |
|  | Reverse | TCAGGGTCCACTTCAAGCAC |  |  |
| ***IFIT2***  *(Interferon Induced Protein With Tetratricopeptide Repeats 2)* | Forward | AGGAAGGGTGGACACGGTTA | 178 | 66,5 |
|  | Reverse | TGCCTCAGAGGGTCAATGGC |  |  |
| ***IFIT3***  *(Interferon Induced Protein With Tetratricopeptide Repeats 3)* | Forward | GGGCAGACTCTCAGATGCTC | 159 | 55 |
|  | Reverse | TCAAAACACACCTTCGCCCT |  |  |
| ***IFITM3***  *(Interferon Induced Transmembrane Protein 3)* | Forward | TAGGGACAGGAAGATGGTTGG | 122 | 54 |
|  | Reverse | GGATGACGATGAGCAGAATGG |  |  |
| ***IRF1***  *(Interferon Regulatory Factor 1)* | Forward | AAGGAAAGTGGGGTCCTTCG | 124 | 67 |
|  | Reverse | ATGTGGCAAGATCCACACGA |  |  |
| ***IRF9***  *(Interferon Regulatory Factor 9)* | Forward | TCCTCCAGAGCCAGACTACT | 87 | 53 |
|  | Reverse | CAATCCAGGCTTTGCACCTG |  |  |
| ***OAS1***  *(2’-5’-Oligoadenylate Synthetase 1)* | Forward | CTTTGATGCCCTGGGTCAGT | 170 | 58 |
|  | Reverse | TGAGGCTCTTGAGCTTGGTG |  |  |
| ***OAS3***  *(2’-5’-Oligoadenylate Synthetase 3)* | Forward | ACTACAACGCCAAGGACAAGA | 71 | 64 |
|  | Reverse | GATGATAGGCCTGGGCTTCTG |  |  |
| ***SOCS1***  *(Suppressor Of Cytokine Signaling 1)* | Forward | CCCTTCTGTAGGATGGTAGCAC | 95 | 67,5 |
|  | Reverse | GAAGAGGAGGAAGGTTCTGGC |  |  |
| ***TAP2***  *(Transporter 2, ATP Binding Cassette Subfamily B Member)* | Forward | TCGCACAGTGCTGGTGATTG | 132 | 53 |
|  | Reverse | ACCAGGCGGGAATAGAGGT |  |  |
| ***WARS1***  *(Tryptophanyl-TRNA Synthetase 1)* | Forward | ACAGCGACTGCATTGGGAAG | 189 | 53 |
|  | Reverse | GGGCTGGTTTAGGATAGCCG |  |  |
