# Supplementary figures and images for: The puzzling regulation of the interferon signaling system by the p53 tumor suppressor protein
Source: Cell Mol Life Sci. 2025 Jun 13;82(1):233. doi: 10.1007/s00018-025-05763-0 (PMC12165926; doi:10.1007/s00018-025-05763-0)

**Supplementary material**


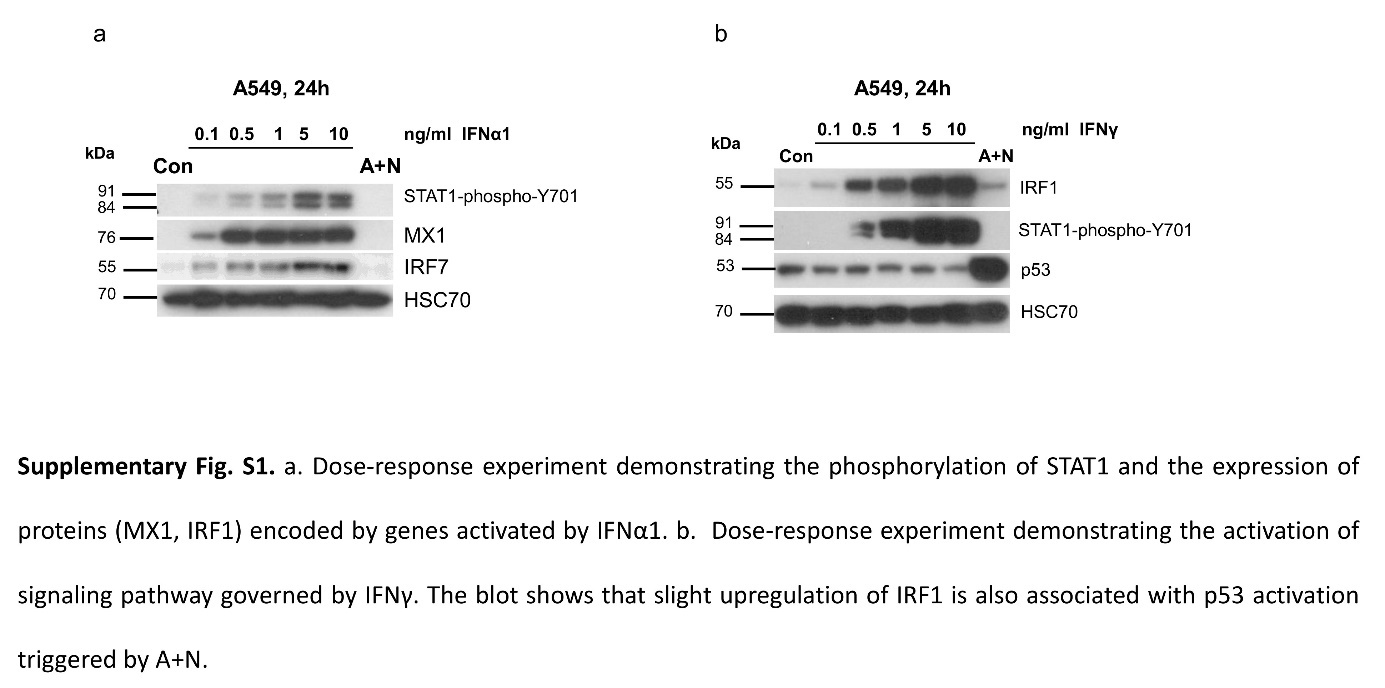


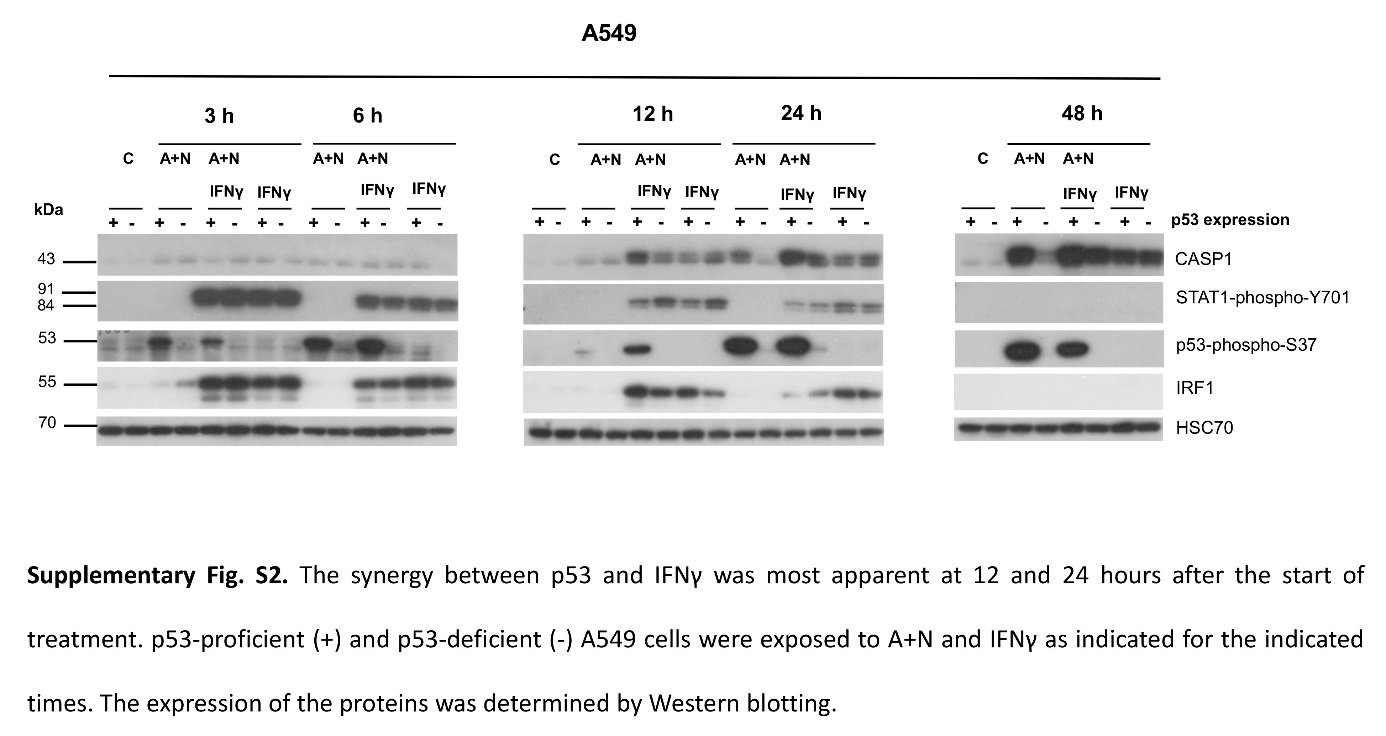


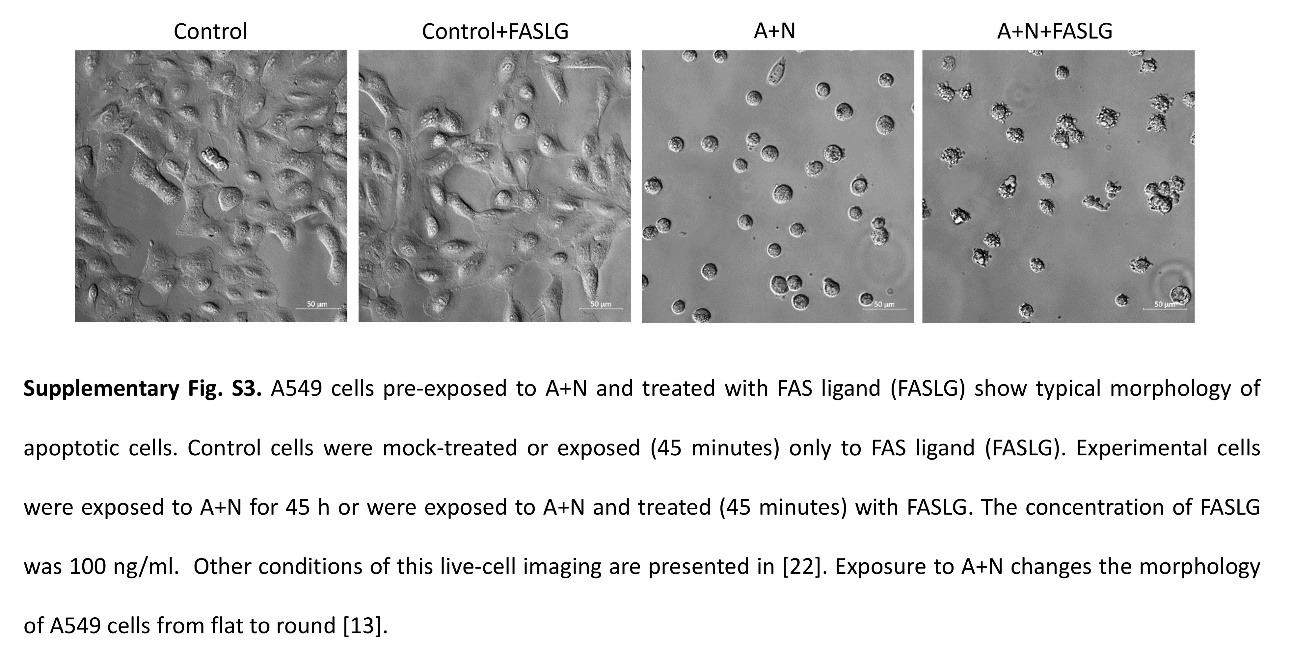

Supplement: Supplementary file 2 — Supplementary Material 2 [file 18_2025_5763_MOESM2_ESM.docx]
